# Tetra­aqua­bis­[*N*,*N*′-bis­(pyridin-3-yl­methyl­idene)benzene-1,4-diamine]­zinc dinitrate 1.49-hydrate

**DOI:** 10.1107/S1600536811046915

**Published:** 2011-11-12

**Authors:** Li Kong, Haihui Yu, Jibo Zhang, Weiyi Cui

**Affiliations:** a Jilin Institute of Chemical Technology, Jilin 132012, People’s Republic of China; b College of Chemical Engineering, Northeast Dianli University, Jilin 132012, People’s Republic of China

## Abstract

In the title compound, [Zn(C_18_H_14_N_4_)_2_(H_2_O)_4_](NO_3_)_2_·1.49H_2_O, the Zn^II^ atom, lying on an inversion center, is coordinated by two N atoms from two *N*,*N*′-bis­(pyridin-3-yl­methyl­idene)benzene-1,4-diamine ligands and four water mol­ecules in a distorted octa­hedral geometry. The nitrate anion is disordered over two sets of sites, with an occupancy ratio of 0.744 (4):0.256 (4). The uncoordinated water mol­ecule is also disordered with an occupancy factor of 0.744 (4). O—H⋯O and O—H⋯N hydrogen bonds link the complex cations, nitrate anions and uncoordinated water mol­ecules into a supra­molecular layer parallel to (102).

## Related literature

For background to the design and synthesis of zinc complexes with Schiff-base ligands and their potential applications as fluorescent probes, see: Su *et al.* (1999 ▶); Ye *et al.* (2005 ▶). For the synthesis of the ligand, see: Ye *et al.* (2004 ▶).
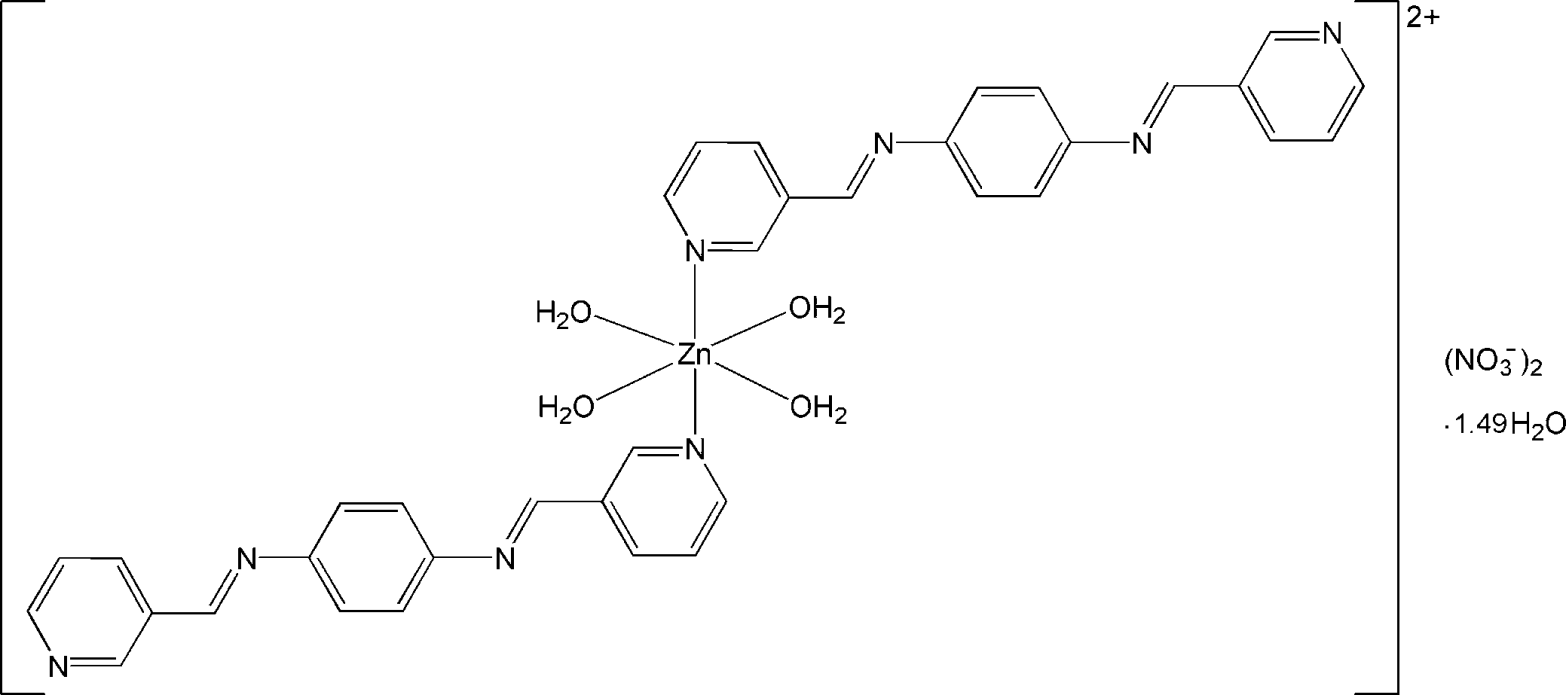


## Experimental

### 

#### Crystal data

[Zn(C_18_H_14_N_4_)_2_(H_2_O)_4_](NO_3_)_2_·1.49H_2_O
*M* *_r_* = 860.95
Triclinic, 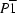

*a* = 8.5664 (17) Å
*b* = 9.928 (2) Å
*c* = 12.496 (3) Å
α = 81.47 (3)°
β = 71.55 (3)°
γ = 78.78 (3)°
*V* = 984.6 (4) Å^3^
*Z* = 1
Mo *K*α radiation
μ = 0.70 mm^−1^
*T* = 295 K
0.48 × 0.28 × 0.18 mm

#### Data collection

Rigaku R-AXIS RAPID diffractometer
Absorption correction: multi-scan (*ABSCOR*; Higashi, 1995 ▶) *T*_min_ = 0.731, *T*_max_ = 0.885
9721 measured reflections
4462 independent reflections
3908 reflections with *I* > 2σ(*I*)
*R*_int_ = 0.019

#### Refinement

*R*[*F* ^2^ > 2σ(*F* ^2^)] = 0.046
*wR*(*F* ^2^) = 0.141
*S* = 1.14
4462 reflections
305 parameters
H-atom parameters constrained
Δρ_max_ = 0.70 e Å^−3^
Δρ_min_ = −0.45 e Å^−3^

### 

Data collection: *RAPID-AUTO* (Rigaku, 1998 ▶); cell refinement: *RAPID-AUTO*; data reduction: *CrystalStructure* (Rigaku/MSC, 2002 ▶); program(s) used to solve structure: *SHELXTL* (Sheldrick, 2008 ▶); program(s) used to refine structure: *SHELXTL*; molecular graphics: *XP* in *SHELXTL* and *Mercury* (Macrae *et al.*, 2006 ▶); software used to prepare material for publication: *SHELXTL*.

## Supplementary Material

Crystal structure: contains datablock(s) I, global. DOI: 10.1107/S1600536811046915/hy2483sup1.cif

Structure factors: contains datablock(s) I. DOI: 10.1107/S1600536811046915/hy2483Isup2.hkl

Additional supplementary materials:  crystallographic information; 3D view; checkCIF report

## Figures and Tables

**Table 1:** Hydrogen-bond geometry (Å, °)

*D*—H⋯*A*	*D*—H	H⋯*A*	*D*⋯*A*	*D*—H⋯*A*
O1*W*—H1*A*⋯O4^i^	0.87	1.87	2.725 (4)	170
O1*W*—H1*A*⋯O4′^i^	0.87	2.23	3.035 (13)	154
O1*W*—H1*B*⋯O3*W*	0.86	2.03	2.859 (13)	161
O1*W*—H1*B*⋯O3′	0.86	1.82	2.65 (3)	161
O2*W*—H2*A*⋯N4^ii^	0.85	1.92	2.706 (3)	152
O2*W*—H2*B*⋯O3^iii^	0.86	1.96	2.761 (3)	155
O3*W*—H3*A*⋯O3^iv^	0.88	2.36	3.073 (12)	139
O3*W*—H3*A*⋯O5^iv^	0.88	2.38	3.112 (13)	142
O3*W*—H3*B*⋯O4	0.88	1.95	2.824 (13)	169

Symmetry codes: (i) 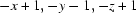; (ii) 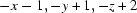; (iii) 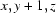; (iv) 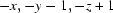.

**Fig. 1.**
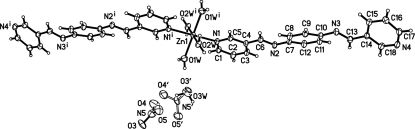
Molecular structure of the title compound. Displacement ellipsoids are drawn at the 30% probability level. H atoms have been omitted for clarity. [Symmetry code: (i) 1 - x, -y, 1 - z.]

**Fig. 2.**
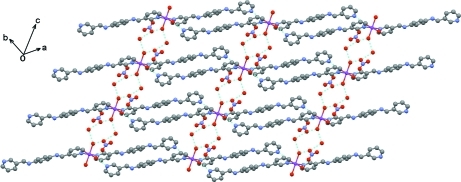
A view of the layer structure in the title compound. Dashed lines denote hydrogen bonds. H atoms and minor disordered nitrate are omitted for clarity.

## supplementary crystallographic information

### Comment

Bipyridine-type ligands have been extensively investigated in recent years, owing to their simple structures, readily availabilities and predictable formation of network structures. Moreover, when introduced in double Schiff-base, a great deal of metal–organic frameworks with unusual network patterns and novel properties can be achieved due to the specific geometry including the different relative orientation of N-donors and the zigzag conformation of the space moiety between the two terminal coordination groups. For background to the design and syntheses of zinc complexes with Schiff-base and their potential applications as fluorescent probes, see: Su *et al.* (1999); Ye *et al.* (2005).

In the title compound (Fig. 1), the Zn^II^ ion lies on an inversion center and is coordinated in a distorted octahedral geometry by two N atoms from two *N*,*N*'-bis(3-pyridylmethylene)-*p*-phenylenediamine (*L*) ligands in the axial positions and four O atoms of four coordinated water molecules in the equatorial positions. The Zn—O distances are 2.0705 (17) and 2.1691 (19) Å and the Zn—N distance is 2.1462 (19) Å. As shown in Fig. 2, the complex cations, nitrate anions and uncoordinated water molecules are connected by O—H···O hydrogen bonds (Table 1), forming a layer structure.

### Experimental

The ligand *L* was prepared according to the previous method (Ye *et al.*, 2004). 1,4-Diaminobenzene (2.14 mg, 10 mmol) was dissolved in methanol (20 ml), followed by addition of 3-pyridinecarboxaldehyde (4.24 mg, 40 mmol). The mixture was stirred at room temperature for 2 h and then filtered. The resulting yellow crystalline solid was washed with methanol several times and dried in air. A solution of Zn(NO_3_)_2_ (35.9 mg, 0.2 mmol) in acetonitrile (10 ml) was slowly layered onto a solution of *L* (117 mg, 0.625 mmol) in methylene chloride (12 ml). Diffusion between the two phases over two weeks produced colorless crystals of the title compound.

### Refinement

H atoms bound to C atoms were positioned geometrically and refined as riding atoms, with C—H = 0.93 Å and with *U*_iso_(H) = 1.2*U*_eq_(C). The water H atoms were located from difference Fourier maps and refined as riding atoms, with *U*_iso_(H) = 1.5*U*_eq_(O). The nitrate anion is disordered over two sets of sites. The occupancy factors were refined to a ratio of 0.744 (4):0.256 (4). The uncoordinated water molecule is also disordered with an occupancy factor of 0.744 (4).

### Crystal data

**Table:**

[Zn(C_18_H_14_N_4_)_2_(H_2_O)_4_](NO_3_)_2_·1.49H_2_O	*Z* = 1
*M**_r_* = 860.95	*F*(000) = 447
Triclinic, *P*1	*D*_x_ = 1.452 Mg m^−^^3^
Hall symbol: -P 1	Mo *K*α radiation, λ = 0.71073 Å
*a* = 8.5664 (17) Å	Cell parameters from 3864 reflections
*b* = 9.928 (2) Å	θ = 3.0–27.5°
*c* = 12.496 (3) Å	µ = 0.70 mm^−^^1^
α = 81.47 (3)°	*T* = 295 K
β = 71.55 (3)°	Block, colorless
γ = 78.78 (3)°	0.48 × 0.28 × 0.18 mm
*V* = 984.6 (4) Å^3^	

### Data collection

**Table:**

Rigaku R-AXIS RAPID diffractometer	4462 independent reflections
Radiation source: rotation anode	3908 reflections with *I* > 2σ(*I*)
graphite	*R*_int_ = 0.019
ω scan	θ_max_ = 27.5°, θ_min_ = 3.0°
Absorption correction: multi-scan (*ABSCOR*; Higashi, 1995)	*h* = −10→11
*T*_min_ = 0.731, *T*_max_ = 0.885	*k* = −12→12
9721 measured reflections	*l* = −16→16

### Refinement

**Table:**

Refinement on *F*^2^	Primary atom site location: structure-invariant direct methods
Least-squares matrix: full	Secondary atom site location: difference Fourier map
*R*[*F*^2^ > 2σ(*F*^2^)] = 0.046	Hydrogen site location: inferred from neighbouring sites
*wR*(*F*^2^) = 0.141	H-atom parameters constrained
*S* = 1.14	*w* = 1/[σ^2^(*F*_o_^2^) + (0.0912*P*)^2^ + 0.1378*P*] where *P* = (*F*_o_^2^ + 2*F*_c_^2^)/3
4462 reflections	(Δ/σ)_max_ = 0.001
305 parameters	Δρ_max_ = 0.70 e Å^−^^3^
0 restraints	Δρ_min_ = −0.45 e Å^−^^3^

### Fractional atomic coordinates and isotropic or equivalent isotropic displacement parameters (Å^2^)

**Table:**

	*x*	*y*	*z*	*U*_iso_*/*U*_eq_	Occ. (<1)
Zn1	0.5000	0.0000	0.5000	0.03476 (14)	
O1W	0.4583 (2)	−0.21267 (19)	0.52697 (18)	0.0542 (5)	
H1A	0.5380	−0.2798	0.5318	0.081*	
H1B	0.3719	−0.2483	0.5325	0.081*	
O2W	0.27473 (19)	0.06743 (19)	0.46591 (14)	0.0442 (4)	
H2A	0.1756	0.0542	0.5011	0.066*	
H2B	0.2754	0.1495	0.4331	0.066*	
N1	0.3790 (2)	0.0222 (2)	0.67673 (15)	0.0366 (4)	
N2	−0.0343 (3)	0.2800 (2)	0.95880 (17)	0.0434 (4)	
N3	−0.5097 (3)	0.7453 (2)	1.10197 (19)	0.0440 (5)	
N4	−0.9932 (2)	0.9640 (2)	1.36277 (19)	0.0471 (5)	
C1	0.4027 (3)	−0.0800 (2)	0.7552 (2)	0.0424 (5)	
H1	0.4803	−0.1575	0.7319	0.051*	
C2	0.3184 (3)	−0.0765 (3)	0.8683 (2)	0.0483 (6)	
H2	0.3395	−0.1497	0.9202	0.058*	
C3	0.2017 (3)	0.0377 (3)	0.9038 (2)	0.0446 (5)	
H3	0.1406	0.0412	0.9798	0.054*	
C4	0.1767 (3)	0.1469 (2)	0.82479 (19)	0.0363 (4)	
C5	0.2690 (3)	0.1342 (2)	0.71269 (19)	0.0375 (5)	
H5	0.2539	0.2074	0.6593	0.045*	
C6	0.0548 (3)	0.2709 (2)	0.8576 (2)	0.0408 (5)	
H6	0.0432	0.3437	0.8031	0.049*	
C7	−0.1540 (3)	0.3988 (2)	0.9907 (2)	0.0399 (5)	
C8	−0.2182 (3)	0.4937 (3)	0.9165 (2)	0.0458 (5)	
H8	−0.1824	0.4822	0.8395	0.055*	
C9	−0.3352 (3)	0.6051 (3)	0.9566 (2)	0.0458 (5)	
H9	−0.3769	0.6686	0.9059	0.055*	
C10	−0.3921 (3)	0.6249 (2)	1.0708 (2)	0.0400 (5)	
C11	−0.3289 (3)	0.5300 (3)	1.1453 (2)	0.0474 (6)	
H11	−0.3652	0.5418	1.2222	0.057*	
C12	−0.2112 (3)	0.4169 (3)	1.1054 (2)	0.0477 (6)	
H12	−0.1704	0.3526	1.1562	0.057*	
C13	−0.6147 (3)	0.7459 (3)	1.1989 (2)	0.0454 (5)	
H13	−0.6136	0.6676	1.2499	0.054*	
C14	−0.7385 (3)	0.8685 (2)	1.2324 (2)	0.0391 (5)	
C15	−0.7352 (3)	0.9931 (3)	1.1659 (2)	0.0443 (5)	
H15	−0.6479	1.0040	1.1000	0.053*	
C16	−0.8622 (3)	1.1002 (3)	1.1987 (3)	0.0511 (6)	
H16	−0.8623	1.1849	1.1554	0.061*	
C17	−0.9892 (3)	1.0806 (3)	1.2962 (2)	0.0473 (6)	
H17	−1.0768	1.1528	1.3164	0.057*	
C18	−0.8701 (3)	0.8603 (3)	1.3314 (2)	0.0458 (5)	
H18	−0.8719	0.7780	1.3780	0.055*	
O3	0.1857 (5)	−0.6809 (2)	0.3557 (3)	0.0680 (9)	0.744 (4)
O4	0.3062 (5)	−0.5772 (4)	0.4302 (5)	0.1089 (17)	0.744 (4)
O5	0.1009 (5)	−0.4680 (3)	0.3743 (3)	0.0796 (10)	0.744 (4)
N5	0.1921 (5)	−0.5781 (4)	0.3878 (3)	0.0653 (10)	0.744 (4)
O3'	0.205 (2)	−0.352 (3)	0.587 (3)	0.063 (4)	0.256 (4)
O4'	0.3410 (16)	−0.4995 (13)	0.4725 (14)	0.109 (5)	0.256 (4)
O5'	0.1218 (17)	−0.5612 (10)	0.5901 (10)	0.091 (4)	0.256 (4)
N5'	0.2171 (12)	−0.4742 (8)	0.5525 (8)	0.050 (2)	0.256 (4)
O3W	0.1962 (15)	−0.3745 (12)	0.5856 (14)	0.108 (4)	0.744 (4)
H3A	0.0988	−0.3961	0.6259	0.162*	0.744 (4)
H3B	0.2440	−0.4359	0.5361	0.162*	0.744 (4)

### Atomic displacement parameters (Å^2^)

**Table:**

	*U*^11^	*U*^22^	*U*^33^	*U*^12^	*U*^13^	*U*^23^
Zn1	0.02806 (19)	0.0387 (2)	0.0302 (2)	0.00313 (13)	−0.00152 (13)	−0.00763 (13)
O1W	0.0454 (10)	0.0420 (9)	0.0700 (13)	−0.0046 (8)	−0.0107 (9)	−0.0064 (8)
O2W	0.0275 (7)	0.0564 (10)	0.0400 (9)	0.0010 (7)	−0.0030 (6)	−0.0030 (7)
N1	0.0319 (8)	0.0402 (9)	0.0307 (9)	0.0030 (7)	−0.0031 (7)	−0.0066 (7)
N2	0.0399 (10)	0.0426 (10)	0.0383 (10)	0.0044 (8)	−0.0015 (8)	−0.0109 (8)
N3	0.0373 (10)	0.0410 (10)	0.0473 (11)	0.0046 (8)	−0.0056 (9)	−0.0138 (9)
N4	0.0330 (9)	0.0576 (12)	0.0448 (11)	−0.0024 (9)	−0.0009 (8)	−0.0164 (10)
C1	0.0356 (11)	0.0429 (11)	0.0392 (12)	0.0066 (9)	−0.0045 (9)	−0.0062 (9)
C2	0.0481 (13)	0.0498 (13)	0.0366 (12)	0.0048 (11)	−0.0082 (10)	0.0020 (10)
C3	0.0405 (12)	0.0538 (13)	0.0302 (11)	0.0032 (10)	−0.0025 (9)	−0.0065 (10)
C4	0.0303 (10)	0.0408 (11)	0.0331 (11)	0.0005 (9)	−0.0035 (8)	−0.0098 (9)
C5	0.0334 (10)	0.0390 (11)	0.0339 (11)	0.0023 (9)	−0.0054 (8)	−0.0055 (9)
C6	0.0380 (11)	0.0404 (11)	0.0372 (11)	0.0023 (9)	−0.0042 (9)	−0.0094 (9)
C7	0.0342 (10)	0.0392 (11)	0.0387 (12)	0.0015 (9)	−0.0017 (9)	−0.0101 (9)
C8	0.0437 (12)	0.0512 (13)	0.0331 (11)	0.0026 (11)	−0.0020 (10)	−0.0090 (10)
C9	0.0405 (12)	0.0474 (12)	0.0414 (13)	0.0039 (10)	−0.0069 (10)	−0.0049 (10)
C10	0.0314 (10)	0.0376 (11)	0.0445 (12)	0.0002 (9)	−0.0028 (9)	−0.0096 (9)
C11	0.0468 (13)	0.0498 (13)	0.0378 (12)	0.0052 (11)	−0.0048 (10)	−0.0144 (10)
C12	0.0477 (13)	0.0480 (13)	0.0368 (12)	0.0082 (11)	−0.0054 (10)	−0.0082 (10)
C13	0.0371 (11)	0.0412 (12)	0.0501 (14)	0.0006 (10)	−0.0048 (10)	−0.0069 (10)
C14	0.0297 (10)	0.0430 (11)	0.0412 (12)	0.0007 (9)	−0.0056 (9)	−0.0126 (9)
C15	0.0384 (11)	0.0486 (13)	0.0405 (12)	−0.0059 (10)	−0.0020 (10)	−0.0104 (10)
C16	0.0504 (14)	0.0402 (12)	0.0574 (16)	−0.0018 (11)	−0.0100 (12)	−0.0083 (11)
C17	0.0357 (11)	0.0463 (12)	0.0556 (15)	0.0028 (10)	−0.0066 (10)	−0.0192 (11)
C18	0.0371 (11)	0.0501 (13)	0.0424 (13)	−0.0031 (10)	−0.0032 (10)	−0.0040 (10)
O3	0.117 (3)	0.0299 (12)	0.0700 (19)	0.0042 (14)	−0.0529 (19)	−0.0107 (11)
O4	0.087 (3)	0.077 (3)	0.184 (5)	0.014 (2)	−0.074 (3)	−0.039 (3)
O5	0.096 (2)	0.0527 (16)	0.082 (2)	0.0106 (16)	−0.031 (2)	−0.0014 (15)
N5	0.071 (2)	0.056 (2)	0.065 (2)	−0.0079 (17)	−0.0215 (18)	0.0064 (16)
O3'	0.046 (6)	0.033 (5)	0.112 (12)	−0.003 (5)	−0.012 (6)	−0.040 (6)
O4'	0.085 (8)	0.088 (8)	0.147 (12)	−0.017 (6)	−0.003 (8)	−0.055 (8)
O5'	0.132 (10)	0.050 (5)	0.087 (8)	−0.030 (6)	−0.022 (7)	0.002 (5)
N5'	0.067 (6)	0.022 (4)	0.058 (5)	0.013 (4)	−0.022 (5)	−0.012 (3)
O3W	0.118 (6)	0.082 (7)	0.137 (6)	−0.035 (4)	−0.028 (4)	−0.043 (5)

### Geometric parameters (Å, °)

**Table:**

Zn1—N1	2.1462 (19)	C8—C9	1.376 (3)
Zn1—O1W	2.1691 (19)	C8—H8	0.9300
Zn1—O2W	2.0705 (17)	C9—C10	1.385 (4)
O1W—H1A	0.8670	C9—H9	0.9300
O1W—H1B	0.8608	C10—C11	1.381 (4)
O2W—H2A	0.8497	C11—C12	1.391 (3)
O2W—H2B	0.8565	C11—H11	0.9300
N1—C1	1.335 (3)	C12—H12	0.9300
N1—C5	1.341 (3)	C13—C14	1.466 (3)
N2—C6	1.259 (3)	C13—H13	0.9300
N2—C7	1.418 (3)	C14—C15	1.384 (4)
N3—C13	1.259 (3)	C14—C18	1.389 (3)
N3—C10	1.420 (3)	C15—C16	1.372 (3)
N4—C17	1.321 (4)	C15—H15	0.9300
N4—C18	1.328 (3)	C16—C17	1.371 (4)
C1—C2	1.371 (3)	C16—H16	0.9300
C1—H1	0.9300	C17—H17	0.9300
C2—C3	1.382 (3)	C18—H18	0.9300
C2—H2	0.9300	O3—N5	1.167 (4)
C3—C4	1.386 (3)	O4—N5	1.252 (5)
C3—H3	0.9300	O5—N5	1.237 (5)
C4—C5	1.384 (3)	O3'—N5'	1.32 (3)
C4—C6	1.468 (3)	O4'—N5'	1.220 (15)
C5—H5	0.9300	O5'—N5'	1.243 (14)
C6—H6	0.9300	O3W—H3A	0.8756
C7—C8	1.383 (4)	O3W—H3B	0.8812
C7—C12	1.388 (3)		
			
O2W—Zn1—O2W^i^	180.0	C4—C6—H6	119.6
O2W—Zn1—N1	90.18 (7)	C8—C7—C12	119.0 (2)
O2W^i^—Zn1—N1	89.82 (7)	C8—C7—N2	124.7 (2)
O2W—Zn1—N1^i^	89.82 (7)	C12—C7—N2	116.3 (2)
O2W^i^—Zn1—N1^i^	90.18 (7)	C9—C8—C7	120.0 (2)
N1—Zn1—N1^i^	180.0	C9—C8—H8	120.0
O2W—Zn1—O1W^i^	88.56 (8)	C7—C8—H8	120.0
O2W^i^—Zn1—O1W^i^	91.44 (8)	C8—C9—C10	121.5 (2)
N1—Zn1—O1W^i^	90.24 (8)	C8—C9—H9	119.3
N1^i^—Zn1—O1W^i^	89.76 (8)	C10—C9—H9	119.3
O2W—Zn1—O1W	91.44 (8)	C11—C10—C9	118.7 (2)
O2W^i^—Zn1—O1W	88.56 (8)	C11—C10—N3	124.8 (2)
N1—Zn1—O1W	89.76 (8)	C9—C10—N3	116.5 (2)
N1^i^—Zn1—O1W	90.24 (8)	C10—C11—C12	120.1 (2)
O1W^i^—Zn1—O1W	180.0	C10—C11—H11	119.9
Zn1—O1W—H1A	120.9	C12—C11—H11	119.9
Zn1—O1W—H1B	131.5	C7—C12—C11	120.7 (2)
H1A—O1W—H1B	107.6	C7—C12—H12	119.7
Zn1—O2W—H2A	133.0	C11—C12—H12	119.7
Zn1—O2W—H2B	108.5	N3—C13—C14	120.9 (2)
H2A—O2W—H2B	110.5	N3—C13—H13	119.6
C1—N1—C5	117.18 (19)	C14—C13—H13	119.6
C1—N1—Zn1	120.75 (15)	C15—C14—C18	117.5 (2)
C5—N1—Zn1	121.93 (15)	C15—C14—C13	122.4 (2)
C6—N2—C7	121.0 (2)	C18—C14—C13	120.0 (2)
C13—N3—C10	120.0 (2)	C16—C15—C14	119.1 (2)
C17—N4—C18	117.9 (2)	C16—C15—H15	120.4
N1—C1—C2	123.4 (2)	C14—C15—H15	120.4
N1—C1—H1	118.3	C17—C16—C15	119.0 (2)
C2—C1—H1	118.3	C17—C16—H16	120.5
C1—C2—C3	118.8 (2)	C15—C16—H16	120.5
C1—C2—H2	120.6	N4—C17—C16	123.2 (2)
C3—C2—H2	120.6	N4—C17—H17	118.4
C2—C3—C4	119.2 (2)	C16—C17—H17	118.4
C2—C3—H3	120.4	N4—C18—C14	123.3 (2)
C4—C3—H3	120.4	N4—C18—H18	118.4
C5—C4—C3	117.6 (2)	C14—C18—H18	118.4
C5—C4—C6	120.7 (2)	O3—N5—O5	123.6 (4)
C3—C4—C6	121.6 (2)	O3—N5—O4	117.9 (4)
N1—C5—C4	123.7 (2)	O5—N5—O4	118.3 (4)
N1—C5—H5	118.2	O4'—N5'—O5'	118.7 (10)
C4—C5—H5	118.2	O4'—N5'—O3'	112.3 (15)
N2—C6—C4	120.8 (2)	O5'—N5'—O3'	129.0 (15)
N2—C6—H6	119.6	H3A—O3W—H3B	107.9

Symmetry codes: (i) −*x*+1, −*y*, −*z*+1.

### Hydrogen-bond geometry (Å, °)

**Table:**

*D*—H···*A*	*D*—H	H···*A*	*D*···*A*	*D*—H···*A*
O1W—H1A···O4^ii^	0.87	1.87	2.725 (4)	170
O1W—H1A···O4'^ii^	0.87	2.23	3.035 (13)	154
O1W—H1B···O3W	0.86	2.03	2.859 (13)	161
O1W—H1B···O3'	0.86	1.82	2.65 (3)	161
O2W—H2A···N4^iii^	0.85	1.92	2.706 (3)	152
O2W—H2B···O3^iv^	0.86	1.96	2.761 (3)	155
O3W—H3A···O3^v^	0.88	2.36	3.073 (12)	139
O3W—H3A···O5^v^	0.88	2.38	3.112 (13)	142
O3W—H3B···O4	0.88	1.95	2.824 (13)	169

Symmetry codes: (ii) −*x*+1, −*y*−1, −*z*+1; (iii) −*x*−1, −*y*+1, −*z*+2; (iv) *x*, *y*+1, *z*; (v) −*x*, −*y*−1, −*z*+1.
